# Water kefir grain polysaccharides: Ultrasonic-assisted extraction optimization, structural characterization, bioactivities, and application in goat yogurt

**DOI:** 10.1016/j.ultsonch.2026.107827

**Published:** 2026-03-18

**Authors:** Wenjuan Zhang, Guowei Shu, Zongcai Zhang, Ting Li, Huan Lei, Huayang Xue, Zhi Wang, Xiaolin Yao, Guoliang Li

**Affiliations:** a School of Food Science and Engineering, Shaanxi University of Science and Technology, Xi’an 710021, China; b Xi’an Baiyue Gaot Milk Corp., Ltd., Xi’an 710089, China; c Shaanxi Youlishi Dairy Group Co., Ltd., Xianyang 713300, China

**Keywords:** Water kefirgrains, Polysaccharides, Ultrasound-assisted extraction, Structural characterization, Biological activities

## Abstract

Polysaccharides from water kefir grains (WG) are functional, food-safe, and potential novel materials for functional food development. This study focused on the extraction process, structural characteristics, and *in vitro* biological activities of polysaccharides from WG (WPU), as well as the effects of WPU on goat yogurt (GY). WPU was optimally extracted from WG via ultrasound-assisted extraction (UE) under the conditions: 340 W (ultrasonic power), 42 min (ultrasonic time), 20 mL/g (liquid-to-solid ratio), and 80 °C (ultrasonic temperature), achieving a high yield of 27.64%. The monosaccharide composition of WPU-4 (the main purified fraction) was glucose (96.59 mol%), arabinose (0.23 mol%), galactose (0.66 mol%), and mannose (2.52 mol%). Its backbone was predominantly composed of 6-Glcp. Scanning electron microscopy (SEM) revealed that WPU-4 exhibited a sheet-like structure, with an uneven and loose porous network on its surface and a honeycomb-like morphology in its interior. *In vitro* assays showed WPU had superior antioxidant, α-glucosidase, and pancreatic lipase inhibitory activities compared to purified fractions. Adding 1.0 mg/mL WPU to GY enhanced its antioxidant, antidiabetic, and hypolipidemic activities. This research provides an efficient WPU extraction method and confirms bioactivity potential, offering technical support for WPU industrial applications as functional ingredients in foods and pharmaceuticals.

## Graphical abstract

**Figure:**
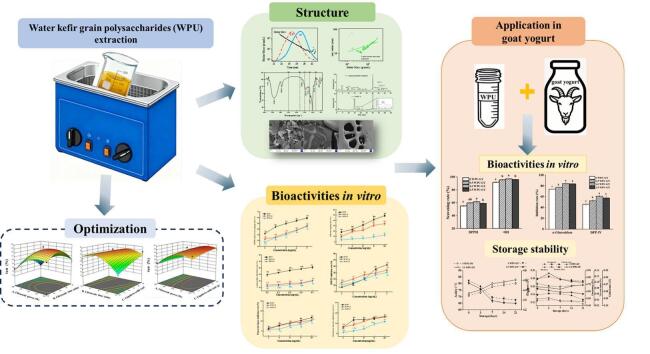

## 1 Introduction

Given their advantages of safety, high bioavailability, and stability, polysaccharides have garnered great concern in the medical and food domains [1]. Among various polysaccharide types, bacterial exopolysaccharides (EPS) secreted by bacteria exhibit unique functions due to their complex and diverse structures. For instance, EPS produced by lactic acid bacteria (LAB) not only serve as stabilizers, thickeners, gelling agents, and emulsifiers in fermented foods [2], but also display diverse bioactivities, including antioxidant, antibacterial, cholesterol-lowering, and antiviral functions [3]. Water kefir grains (WG) with a cauliflower-like appearance contain yeasts, bacteria, and bacterial EPS [4]. Among the bacterial species, LAB predominate besides acetic acid bacteria and bifidobacteria [5]. Most available studies on WG have focused on its application in producing homemade fermented beverages [6]. There are a few reports on the polysaccharides generated by the WG. Tan et al. [4] reported that polysaccharides produced by *Liquorilactobacillus satsumensis* (derived from WG) had prebiotic properties. Moreover, Fels et al. [5] found that the EPS obtained from the whole WG is produced with the participation of a variety of bacteria. Lucena et al. [7] demonstrated that the EPS obtained from whole WG by hot water extraction (HE) exhibited excellent emulsifying properties, cytocompatibility, and exerted antibacterial effects against *Staphylococcus aureus* and *Escherichia coli* as well. Therefore, WG polysaccharides as novel food functional materials exhibit extensive application prospects.

Additionally, HE remains the primary method for extracting WG polysaccharides. However, the HE method depends on elevated temperatures and extended extraction conditions; such drastic conditions not only reduce polysaccharide yield but also impair their bioactivity via thermal degradation, resulting in the fragmentation of their molecular chains [8]. Ultrasound treatment has been extensively applied in polysaccharide extraction owing to its advantages of safety, high efficiency, and energy conservation. Ultrasound, triggering a transient cavitation effect in the extraction medium, breaks down the cell walls and facilitates the efficient liberation of polysaccharides [8]. Compared with HE, ultrasonic-assisted extraction (UE) has advantages in both the preservation of polysaccharides' biological activity and the efficiency of extraction. For instance, UE of Hawk tea polysaccharides (UHTP) increased the extraction yield by 17.00% compared with HE, and UHTP exhibited stronger antioxidant, hypoglycemic, and prebiotic activities [9]. Similarly, the yield of *Morus alba* mycelium polysaccharides extracted via UE increased by 87.5% compared to HE [10]. Notably, goat milk possesses unique physiological and biochemical properties, which often make it superior to bovine milk [11]. Moreover, goat yogurt (GY) is recognized as a favorable option for potential consumers who have demands for distinctive flavors, strengthened health benefits, or substitutes for bovine milk to address allergen concerns [12]. Previous research has revealed that polysaccharides could enhance the physiological functions of fermented milk, such as antioxidant [13] and antidiabetic activities [14]. Nevertheless, the influence of WPU on the biological activities of GY is not yet fully elucidated.

Therefore, this study employed UE for extracting polysaccharides from WG (WPU), and systematically optimized the extraction parameters to develop a high-yield extraction method. After systematic separation and purification of WPU, the potential *in vitro* antioxidant, antidiabetic, and hypolipidemic bioactivities of the main purified fraction were evaluated, along with its structural characterization. Additionally, the influence of incorporating WPU into GY on the bioactive properties and storage characteristics of GY was investigated. The objectives of this research are to provide technical support for the efficient utilization of WG polysaccharides and to build a scientific foundation for their applicability to the food field.

## 2 Materials and methods

### 2.1 Materials and reagents

WG were obtained from a store in Shenyang, Liaoning Province. α-Glucosidase (α-Glu) was purchased from Shanghai Yuanye Biotechnology Co., Ltd. Dipeptidyl peptidase IV (DPP-IV) was obtained from ProSpec-Tany TechnoGene Ltd. (Ness-Ziona, Israel). Cholesterol esterase was obtained from Shanghai Macklin Biochemical Technology Co., Ltd. All other chemicals used in this study were of analytical grade and obtained from Sigma-Aldrich (St. Louis, MO, USA).

### 2.2 UE process

The manufacture of WG was adapted from previous studies [15]. The WG (5 g/100 g) was activated for six days with a 10% (w/v) brown sugar solution. To maintain their fermentative activity and viability, the grains were supplied with fresh brown sugar solution (nutrient source) every 24 h for continuous nutrient exchange.

The polysaccharide extraction method was derived from Lucena et al. [7] with slight modifications. Under a specified liquid-to-solid ratio (LSR) and other given conditions, WG were homogenized (15000 rpm, 5 min) and then extracted in an ultrasonic cleaner. The obtained extract was subjected to high-speed centrifugation (9000 rpm, 15 min), after which the supernatant was retained for subsequent analysis. Trichloroacetic acid (4%, w/v) was then added to this supernatant. After 12 h of incubation at 4 °C, the mixture was subjected to high-speed centrifugation (10000 rpm, 10 min) for the elimination of protein. Absolute ethanol was added to the supernatant at a volume ratio of 3:1 (ethanol: supernatant), and the mixture was incubated (4 °C, 12 h) to precipitate the polysaccharides. The precipitated WG polysaccharides were collected via centrifugation (10000 rpm, 20 min), dissolved in distilled water, followed by 48 h of dialysis with a 1 kDa cut-off. Following freeze-drying treatment, the dialysate was labeled WPU.

### 2.3 Optimization design

The single-factor (SF) experiment plan was designed with the following variables (using WG polysaccharides yield as the evaluation index): ultrasonic temperature (40–80 °C; interval: 10 °C), ultrasonic time (20–60 min; interval: 10 min), ultrasonic power (240–400 W; interval: 40 W), and LSR (10–30 mL/g; interval: 5 mL/g) (Table S1). To explore the effect of each experimental factor on WPU extraction rate, a one-factor-at-a-time approach was adopted, and the effect of each variable on polysaccharide yield was investigated separately.

The polysaccharide extraction conditions were optimized via response surface (RS) methodology, with the yield of polysaccharides selected as the response variable. The optimization factors were defined as ultrasound power (*A*), ultrasound time (*B*), and liquid-to-solid (*C*). The phenol sulfate method was employed for the quantification of polysaccharide content [16]. Extraction yields were calculated by the formula:

(1) $$\text{Yield}\;\left(\%\right)=\left(\text{A}\;\times \;\text{B}\;\times \;\text{C}\right)/\left(\text{1}000\times \;\text{D}\right)\times \;\text{1}00$$

where A represents the measured concentration of polysaccharides (mg/mL), B represents the original volume of filtrate (mL), C represents the dilution ratio, and D represents the weight of WG (g).

### 2.4 Purification

WPU purification was performed based on the report by Deng et al. [17], with appropriate modifications. WPU was separated on a DEAE-52 cellulose column (1.6 cm × 30 cm), with stepwise elution performed using distilled water and graded NaCl solutions (0.05 M, 0.1 M, 0.3 M). Three polysaccharide fractions were obtained (WPU-I, WPU-Ⅱ, WPU-Ⅲ). Subsequently, a dominant independent fraction was separated using ultra-filtration membranes (300, 100, 50, 10, and 5 kDa) to obtain polysaccharide fractions with different molecular weights (Mw), which were then freeze-dried for subsequent analysis.

### 2.5 Characterization

#### 2.5.1 Mw and homogeneity analysis

The Mw and homogeneity of the WG polysaccharides were analyzed following a previously established method [18]. Specifically, detection was performed with a Gel Permeation Chromatography (GPC)-refractive index detector-laser photometer system, which consisted of a U3000 liquid chromatography system (Thermo, USA), an Optilab T-rEX refractive index detector (Wyatt Technology, CA, USA), and a DAWN HELEOS II multi-angle laser light scattering detector (Wyatt Technology, CA, USA). Chromatographic separation was carried out on two Ohpak SB-805 HQ (300 × 8 mm) and Ohpak SB-803 HQ (300 × 8 mm) gel permeation columns connected in series, maintained at 45 °C. Chromatographic conditions: mobile phase (0.1 mol/L NaNO_3_ with 0.02% NaN_3_), flow rate (0.6 mL/min), injection volume (100 μL).

#### 2.5.2 Monosaccharide compositions analysis

Following the method of Wang et al. [19], the monosaccharide composition of WPU-4 was determined. The polysaccharide sample (2 mg) was mixed with a 2 M trifluoroacetic acid (TFA) solution (1 mL) and hydrolyzed (121 °C, 2 h). Then, residual acid was removed by nitrogen blow-drying, followed by rinsing with high-purity methanol and subsequent blow-drying. Finally, the treated samples were dissolved in distilled water for subsequent measurement.

#### 2.5.3 Fourier transform infrared (FTIR) spectroscopy

FTIR spectroscopy (Shimadzu, Japan) was conducted with a scanning range of 4000–400 cm^−1^. During sample preparation, 1 mg of sample powder and 150 mg of dried KBr were compressed into pellets to facilitate the analysis.

#### 2.5.4 Scanning electron microscope (SEM)

The dried WPU-4 was placed on mica sheets before gold-coating treatment. Observation and image acquisition were performed using a SEM (Regulus 8100, Hitachi, Japan) at magnifications of 1000×, 500×, and 200 × under a 5 kV working voltage.

#### 2.5.5 Methylation analysis

Methylation analysis was conducted according to a previous study [20]. WPU-4 was dissolved in a DMSO/NaOH mixed solution, followed by the addition of CH_3_I. The reaction was then incubated in the dark (25 °C, 12 h). Following complete methylation, the methylated polysaccharide was subjected to hydrolysis with 2 mol/L TFA (121 ℃, 1.5 h), reduction using NaBD_4,_ and acetylation using acetic anhydride (100 ℃, 2.5 h). Subsequently, the methylated samples were analyzed by an Agilent 6890A-5977B GC–MS.

### 2.6 Biological activities

#### 2.6.1 Antioxidant activity

Measurement of 2,2-Diphenyl-1-picrylhydrazyl radical (DPPH) scavenging activity (DPPH-SA) was carried out based on the approach reported by Chen et al. [21]. Briefly, 40 μL of polysaccharide solutions with different concentrations (1, 2, 3, 4, and 5 mg/mL) were mixed with 160 μL of DPPH (0.1  mM in 50% ethanol). Absorbance measurement of the mixture was performed at 517 nm.

Polysaccharides were prepared as aqueous solutions at different concentrations ranging from 2 to 10 mg/mL at 2 mg/mL intervals. Hydroxyl radical (·OH) scavenging activity was assessed following the method reported by Guan et al. [18].

#### 2.6.2 Antidiabetic activity

##### 2.6.2.1 α-Glu inhibitory activity

The inhibitory rates of the samples against α-Glu were measured according to the method reported by Chen et al. [22] with a few modifications. A reaction system was established by mixing 25 μL of gradient-concentration polysaccharide solutions (0.1–0.5 mg/mL), 50 μL of α-Glu solution (0.2 U/mL), and 25 μL p-nitro-phenyl-α-D-glucopyranoside solution (2.5 mmol/L). After incubating the resultant mixture at 37 °C for 0.5 h, the enzymatic reaction was quenched by adding Na_2_CO_3_ solution (100 μL, 0.2 mol/L). Absorbance values were measured at 405 nm, and the α-Glu inhibitory rate was computed using the following formula:

(2) $$\alpha -\text{Gluinhibitionrate}\left(\%\right)=\;\text{1}-\left({\text{A}}_{\text{s}}-{\text{A}}_{\text{sc}}\right)/\left({\text{A}}_{\text{c}}-{\text{A}}_{\text{b}}\right)\times \;\text{1}00$$

Wherein, A_s_: absorbance of sample (sample + enzyme + substrate); A_sc_: absorbance of sample control (sample + buffer + substrate); A_c_: absorbance of control (buffer + enzyme + substrate); A_b_: absorbance of blank groups (buffer + substrate).

##### 2.6.2.2 DPP-IV inhibitory activity

The inhibition of the sample against DPP-IV was estimated based on a previous study [23]. Polysaccharide solutions (1–5 mg/mL, 25 μL) were combined with DPP-IV solution (0.01 U/mL, 50 μL) and Gly-Pro-p-nitroanilide substrate (1.6 mmol/L, 25 μL). The reaction mixture was incubated at 37 °C for 1 h. Absorbance readings were taken at 405 nm, and the inhibition rate was calculated using the formula:

(3) $$\text{DPP}-\text{IV inhibition rate}\;\left(\%\right)=\;\text{1}-\left({\text{A}}_{\text{s}}-{\text{A}}_{\text{sc}}\right)/\left({\text{A}}_{\text{c}}-{\text{A}}_{\text{b}}\right)\times \;\text{1}00$$

Wherein, A_s_: sample absorbance; A_sc_: sample control absorbance; A_c_: control absorbance; A_b_: blank group absorbance.

#### 2.6.3 Hypolipidemic activities

##### 2.6.3.1 Pancreatic lipase (PL) inhibitory activity

The PL inhibition rate was assessed using the method described by Zhang et al. [24]. Briefly, 50 μL of polysaccharide solutions (2, 4, 6, 8, and 10 mg/mL) were mixed with PL solution (30 U/mL, 50 μL) and incubated (37 °C, 20 min). Subsequently, p-nitrophenyl butyrate substrate (5 mM, 50 μL) was added to initiate the reaction, which was then incubated for 10 min. Absorbance readings were taken at 405 nm. The PL inhibition rate calculation formula is provided below:

(4) $$\text{PL inhibition rate}\;\left(\%\right)=\;\text{1}-\left({\text{A}}_{\text{s}}-{\text{A}}_{\text{sc}}\right)/\left({\text{A}}_{\text{c}}-{\text{A}}_{\text{b}}\right)\times \;\text{1}00$$

Wherein, A_s_: sample absorbance; A_sc_: sample control absorbance; A_c_: control absorbance; A_b_: blank group absorbance.

##### 2.6.3.2 Cholesterol micelles (MC) inhibitory activity

A method described by Su et al. [25], with minor modifications, was utilized to investigate the effect of polysaccharides on MC. Three volumes of the MC solution were added to polysaccharide solutions (2–10 mg/mL, intervals 2 mg/mL). Following shaking at 37 °C for 1 h, the mixture was subjected to centrifugation (12000 rpm, 20 min); subsequently, the cholesterol content in the supernatant was assayed.

### 2.7 Application of WPU in goat yogurt (GY)

A 12.5% (w/v) goat milk powder solution was prepared, after which WPU was incorporated at various concentrations (0.5–1.5 mg/mL, intervals 0.5 mg/mL), corresponding to the samples labeled 0.5WPU-GY, 1.0WPU-GY, and 1.5WPU-GY. The GY without WPU (0WPU-GY) served as the control. At 90 °C, the mixture was pasteurized for 10 min, with subsequent cooling to 40 °C. After cooling, it was inoculated with a 0.005% (w/v) commercial starter (*Lactobacillus delbrueckii* subsp. *bulgaricus* and Streptococcus salivarius subsp. *thermophilus*) and subsequently incubated (42 °C, 4.5 h) to obtain GY samples.

Determination of textural properties (cohesiveness, viscosity index, consistency, and hardness), titratable acidity (TA), and pH values was conducted using the method reported by Zhang et al. [26]. Additionally, GY was centrifuged to collect whey samples (8000 rpm, 10 min), and the antioxidant (Section 2.6.1), antidiabetic (Section 2.6.2), and hypolipidemic (Section 2.6.3) activities of these whey samples were measured respectively, following the aforementioned methods.

### 2.8 Statistical analysis

All graphical illustrations were generated with Origin 2021. IBM SPSS Statistics 22 was employed for statistical analysis to detect significant variations among groups and samples.

## 3 Results and discussion

### 3.1 SF experiments

**Fig. 1:**
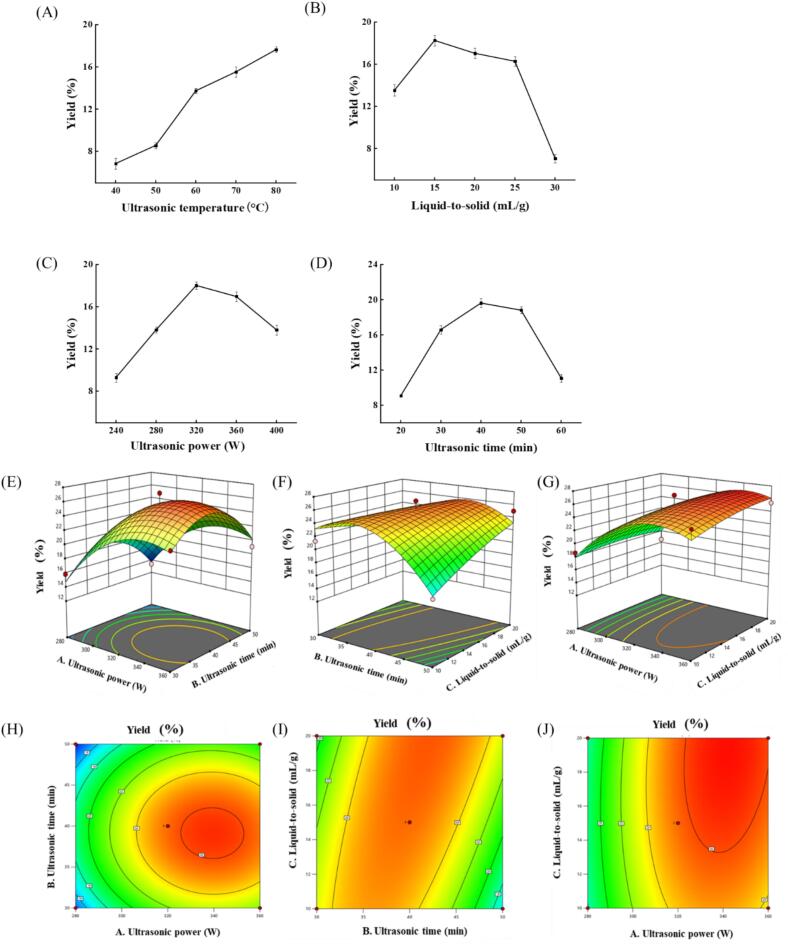
Effect of ultrasonic temperature (A), liquid-to-solid ratio (B), ultrasonic power (C), and ultrasonic time (D) on the yield of WPU; 3D (E-G) and 2D (H-J) response surface and contour plots illustrating the influence of interactions among various factors on the yield of WPU.

Results shown in Fig. 1A demonstrate that ultrasonic treatment temperatures of 40–80 °C were accompanied by a marked enhancement in WPU yield, which increased from 6.84% to 17.64%. This phenomenon might be attributed to the increased temperature, which hastened molecular motion and promoted polysaccharide solubilization [8]. Given that excessively high temperatures might alter the structure of polysaccharides and reduce their biological activities, 80 °C was chosen as the optimum ultrasonic temperature and not considered as a variable. Fig. 1B illustrates the effect of LSR on WPU yield. When the LSR was increased in the range of 10–30 mL/g, the yield of WPU underwent an initial increase and then a decrease, reaching its maximum of 18.26% when the LSR was 15 mL/g. A plausible explanation was that as the LSR elevated, the solubility of other soluble substances increased, which in turn reduced the solubility of polysaccharides [27]. Therefore, 15 mL/g was selected as the optimal LSR to be employed in subsequent experiments.

Fig. 1C presents the relationship between varying ultrasonic power levels and extraction yield. When the ultrasonic power was increased in the range of 240–400 W,

WPU's yield increased initially and subsequently decreased, achieving the highest value at 320 W. It might be because the cell structure was destroyed by the ultrasonic cavitation effect, which would enhance the polysaccharides release. Exceeding the critical value of ultrasonic power, the redundant mechanical effect tended to bring about local overheating, which exerted a negative impact on the stability of polysaccharides [8].

As demonstrated in Fig. 1D, with the prolongation of ultrasonic time (20–60 min), the yield of WPU initially increased and subsequently decreased, achieving its highest value of 19.62% at 40 min. While ultrasound technology accelerated the dissolution and diffusion of substances, long-term use produced free radicals and reactive oxygen species that damaged polysaccharides' chemical bonds, leading to their decomposition and degradation [28]. Thus, the extraction time should be controlled at 40 min.

The results revealed that SF conditions were linked to WPU yield, with a marked reduction in yield observed when certain factors exceeded their respective critical values. While SF experiments presupposed factor independence, most factors were interrelated in practical scenarios [29]. Consequently, additional analysis should be carried out using the results of these SF experiments.

### 3.2 RS experiments

Given the results of the SF experiments, a Box-Behnken design involving three factors and three levels was adopted for further optimization of the extraction process. The independent factors selected were ultrasonic power (*A*), ultrasonic time (*B*), and LSR (*C*), with the yield of WPU chosen as the response variable (R1). Fifteen experiments were performed, with the results tabulated in Table S2. The complex relationship between test variables and response variables was established: Yields (%) = 25.56 + 3.43A-0.679B + 0.660C-0.150AB + 0.568AC + 2.51BC-3.56A^2^-4.35B^2^-0.541C^2^. In the equation, the absolute values of the coefficients directly reflected the intensity of the influence of each factor on the indicator values, with the coefficient signs indicating the direction of this impact [30]. It could be concluded that the sequence of significance of factors impacting the WPU yield was as follows: ultrasonic power (*A*) > ultrasonic temperature (*B*) > LSR (*C*).

Table S3 demonstrates that the linear terms of *A* and *B*^2^ exhibited highly significant regression coefficients (*p* < 0.01), while the linear terms of *BC* and *A*^2^ were significant (*p* < 0.05). However, the coefficients of the remaining variables showed no significance. This indicated that the associations between the various factors were not simple linear relationships, but there were significant interactions among them. Overall analysis revealed the significance of the model (*p* = 0.0224 < 0.05), and the lack-of-fit test resulted in *p* = 0.2387 > 0.05, suggesting the model had a good fitting degree [30]. Furthermore, the model exhibited an R^2^ value of 0.926 > 0.9, further verifying the experimental model’s significance and reliability [31].

The 2D and 3D RS and contour plots are shown in Fig. 1E-I, which illustrates the interactive effects among factors. The surface steepness reflected the extent to which interactions among ultrasonic power, ultrasonic time, and LSR influence the yields. Furthermore, the contour lines' elliptical configuration suggested that the mutual interactions among these factors exerted notable impacts on the yield. As indicated by the model, the optimal conditions were 342.28 W, 42.02 min, and 20 mL/g, with a predicted WPU yield of 26.98%. Given practical application requirements, these conditions were modified to 340 W, 42 min, and 20 mL/g. The average WPU yield of 27.64%±0.92% (27.81%, 25.96%, 27.19%) was achieved from three parallel runs under the optimized conditions, validating the predictive capability of the model.

### 3.3 Isolation and purification of WPU

**Fig. 2:**
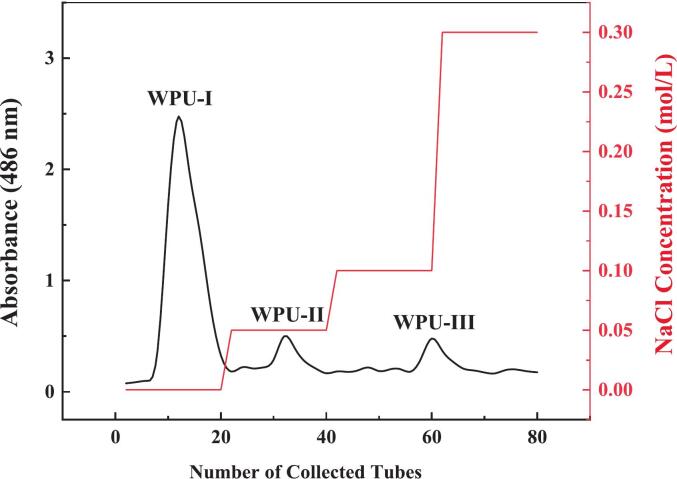
Elution profile of WPU on DEAE-52 column.

DEAE‑cellulose is an effective method for polysaccharide purification. As shown in Fig. 2, after separation via the DEAE-52 column, three main fractions named WPU-Ⅰ, WPU-Ⅱ, and WPU-Ⅲ were isolated. WPU-III was noted at the boundary between 0.1 M and 0.3 M NaCl. A possible reason was that, during stepwise elution, the transient salt gradient formed when switching from 0.1 M to 0.3 M NaCl effectively desorbs WPU-III, leading to elution at this boundary. Yang et al. [32] also observed similar results when isolating *Z. jujuba* cv. *Hamidazao* polysaccharides using DEAE‑cellulose 52. Considering the yield, WPU‑Ⅰ was selected for subsequent experiments.

Ultrafiltration is a novel and efficient method for polysaccharide extraction, enabling the selective filtration, concentration, and purification of polysaccharides [33], [34]. The main components of WPU-I were separated using ultrafiltration membranes with different Mw cut-offs (10 kDa, 50 kDa, 100 kDa, and 300 kDa), obtaining five parts, named WPU-1 (>300 kDa), WPU-2 (100–300 kDa), WPU-3 (50–100 kDa), WPU-4 (10–50 kDa), and WPU-5 (< 10 kDa), respectively. The yield rates of the five component fractions are 23.55%, 2.61%, 7.55%, 35.19%, and 31.10%, respectively. WPU-4 and WPU-5, which exhibited higher yields, were selected for subsequent research.

### 3.4 Antioxidant activity of polysaccharides

**Fig. 3:**
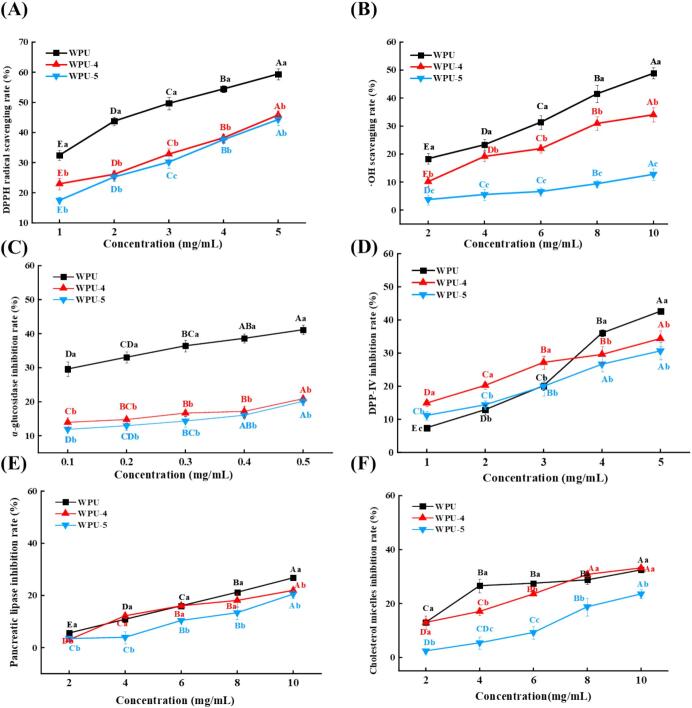
DPPH radical scavenging rates (A), ·OH scavenging rates (B), and inhibition activities of α-glucosidase (C), DPP-Ⅳ (D), pancreatic lipase (E), and cholesterol micellar (F) of WPU, WPU-4, and WPU-5. Note: For the same sample, different uppercase letters represent significant differences among various concentrations; at the same concentration, different lowercase letters represent significant differences among different samples. The statistical significance threshold was set at *p* < 0.05.

The DPPH-SA of WPU, WPU-4, and WPU-5 (WPUs) is shown in Fig. 3A. It could be observed that significantly higher DPPH-SA was detected in WPU compared to WPU-4 and WPU-5 (*p* < 0.05) as the concentration increased from 1 to 5 mg/mL. Under the condition of 5 mg/mL, the DPPH-SA of WPU, WPU-4, and WPU-5 were 59.41%, 45.78%, and 44.31%, respectively. The higher DPPH-SA of WPU was likely attributable to the presence of additional bioactive components, which functioned in synergy with polysaccharides to enhance the DPPH-SA [35]. This observation aligns with the findings of Yang et al. [35], who observed that crude polysaccharides from *Boschniakia rossica* had higher DPPH-SA compared to the purified fractions. Similarly, Guo et al. [36] found that crude polysaccharides exhibited higher DPPH-SA compared to purified longan polysaccharides. At a concentration of 1 mg/mL, the DPPH-SA of WPU-4 was higher than that of WPU-5. This could be attributed to the higher Mw of WPU-4, which provides more hydrogen-donating sites and maintains good solubility at low concentrations, thereby enhancing its DPPH-SA [37].

As shown in Fig. 3B, consistent with the results of DPPH-SA, WPU exhibited a significantly higher ·OH scavenging rate than WPU-4 and WPU-5 (*p* < 0.05), with the increase of concentration (2–10 mg/mL). In addition, compared with WPU-5, WPU-4 showed a significantly higher ·OH scavenging rate (*P* < 0.05). At 10 mg/mL, the ·OH scavenging rates of WPU, WPU-4, and WPU-5 were 48.86%, 34.05%, and 12.76%, respectively. Generally, low-Mw polysaccharides tend to exhibit potent antioxidant activity, due to the higher content of reducing hydroxyl groups at the terminals [32]. However, even polysaccharides isolated from one source can exhibit discrepancies in terms of antioxidant activity, which may be attributed to differences in their monosaccharide composition, chemical structure, chain arrangement, and molecular weight [38]. The study by Su and Li [39] corroborated our experimental outcomes, demonstrating a positive relationship between polysaccharide molecular weight and antioxidant efficacy, which suggested that molecular weight was not the determining factor.

### 3.5 Antidiabetic activity of polysaccharides

Oligosaccharides and disaccharides can be cleaved into monosaccharides through the catalytic action of α-Glu, which leads to an increase in the body’s glucose level [17]. Consequently, the activity of polysaccharides to inhibit α-Glu is commonly employed for assessing their hypoglycemic potential. The inhibition activities of WPU, WPU-4, and WPU-5 against α-Glu are presented in Fig. 3C. With the elevation of concentration (0.1–0.5 mg/mL), the inhibition rates of WPU were consistently significantly higher than those of WPU-4 and WPU-5 (*p* < 0.05). WPU-4 and WPU-5 exhibited no significant difference in α-Glu inhibition (*p* > 0.05). At 0.5 mg/mL, for WPU, WPU-4, and WPU-5, the inhibition rates were 41.16%, 20.88%, and 20.13%, respectively. The α‑Glu inhibition rate of WPU was superior to that of the polysaccharides from wild jujube (WJFP‑W), which was 35.28% [40]. However, it was lower than that of AP‑1 (a neutral polysaccharide from apricot), whose α‑Glu inhibition rate exceeded 60% at 0.5 mg/mL [41]. Possible explanations for the lower α-Glu inhibition of WPU-4 and WPU-5 are that polysaccharides fail to form active conformations when their relative Mw is too low, thereby decreasing their biological activities [42]. In addition, it has also been reported that larger molecules could provide a physical barrier to enzymes, preventing them from interacting with substrates [43]. Additionally, WPU may contain other active components such as polyphenols. Zhu et al. [44] demonstrated that *Hovenia dulcis* polysaccharides–quercetin (HDPs-Q) exhibited significantly greater α-Glu inhibitory activity than polysaccharides alone.

Glucagon-like peptide-1 (GLP-1) is an incretin hormone that contributes to the regulation of metabolic homeostasis and enhances insulin secretion. However, DPP-IV can degrade GLP-1 rapidly. Therefore, DPP-IV inhibition activity is recognized as a key approach for identifying antidiabetic active substances [45]. Fig. 3D characterizes the DPP-IV inhibitory activities of WPU, WPU-4, and WPU-5 across a concentration range from 1 to 5 mg/mL. Overall, the abilities of WPU, WPU-4, and WPU-5 to inhibit DPP-IV activity improved with increasing concentration. Evidently, the DPP-IV inhibition rate could be determined as WPU-4 > WPU-5 > WPU, at 1 mg/mL. Thambi and Chakraborty [46] reported that after purification of the polysaccharides from *Hydropuntia edulis*, the purified fraction SP-He-2 displayed a remarkably enhanced DPP-IV inhibition activity compared with the crude polysaccharide. However, at 5 mg/mL, WPU showed the highest DPP-IV inhibition rate. This may be attributed to the complex structure of high-Mw polysaccharides in WPU, which contributes to their antidiabetic activity [47].

### 3.6 Hypolipidemic activity of polysaccharides

PL can digest approximately 50%-70% of consumed lipids, as it catalyzes the hydrolysis of triglycerides into glycerol and free fatty acids. Therefore, inhibiting PL activity represents a pivotal strategy for regulating cholesterol and lipid metabolism [48]. Fig. 3E shows the inhibition rates of WPUs against PL. At a 10 mg/mL concentration, WPU exhibited the highest inhibition rate (*p* < 0.05), with a value of 26.75%, while those of WPU-4 and WPU-5 were 21.94% and 20.51%, respectively. The reason for this phenomenon might be the presence of higher molecular weight polysaccharides in the WPU. A previous study suggested that polysaccharides with high molecular weight were more potent enzyme inhibitors, potentially because their bulkier structure sterically hindered the enzyme's active site, thereby limiting substrate binding [43]. Additionally, the stronger inhibition of PL activity by polysaccharides of higher MW may be attributed to their higher apparent viscosity [49]. Furthermore, compared with previous reports, the inhibitory effects of mulberry leaf polysaccharides (2.5 mg/mL, 20% inhibition) [50] and *Stropharia rugosoannulata* polysaccharides (3.75 mg/mL, 70.68% inhibition) [49] on PL activity were both stronger than those of WPUs. Nevertheless, this study is the first to report the PL inhibitory activity of WPUs. As a naturally derived polysaccharide, WPUs are inherently safe. Although their inhibitory activity is relatively moderate, they provide a novel starting point for developing new natural lipid-lowering functional foods.

The uptake of cholesterol involves its dissolution into complex spherical phospholipid bodies known as micelles, which promote lipid translocation into enterocytes. Therefore, inhibiting cholesterol solubilization into micelles has emerged as a promising target for cholesterol reduction [51]. The MC inhibition of WPU, WPU-4, and WPU-5 is presented in Fig. 3F. At 10 mg/mL, their inhibition rates were 32.48%, 33.20%, and 28.93%, respectively. In conclusion, these results indicate that WPUs possess potential hypolipidemic activity.

### 3.7 Structure characteristic

#### 3.7.1 Molecular weight analysis of WPU-4

**Fig. 4:**
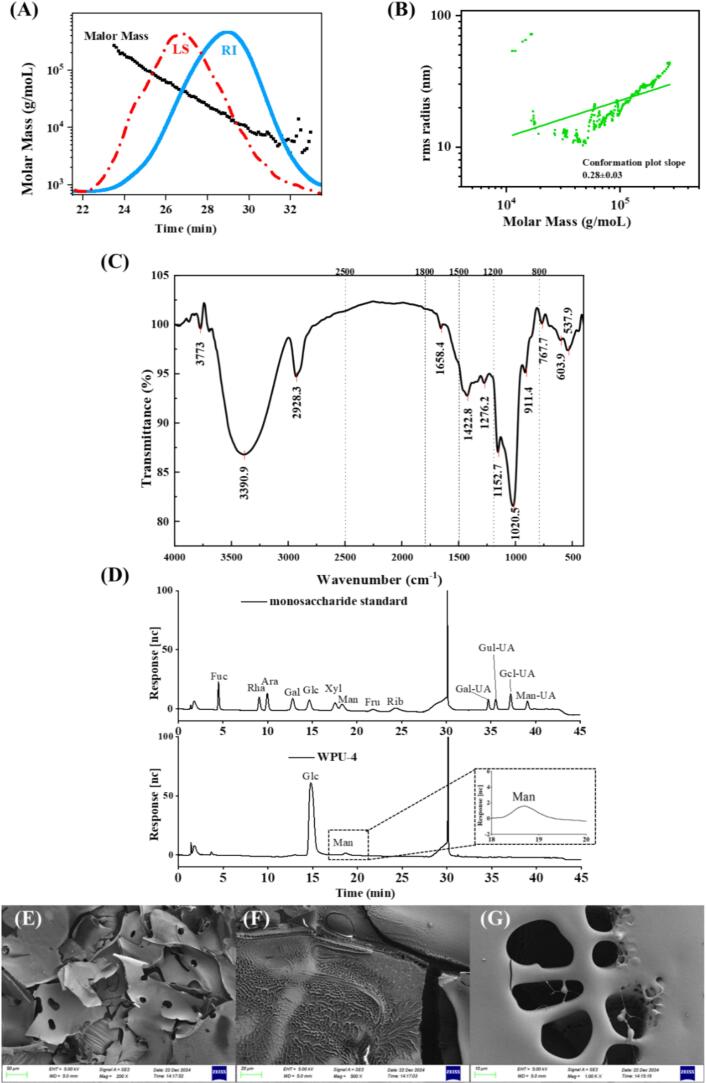
Molecular weight (A), molecular conformation (B), FTIR spectrum (C), monosaccharide composition (D), and SEM images under different magnifications (E: 200×, F: 500×, G:1000 × ) of WPU-4.

Based on the aforementioned research results, WPU-4 was selected for subsequent structural investigations. As depicted in Fig. 4A, the chromatograms of light scattering (LS) and refractive index (RI) both exhibited a single symmetric and narrow peak, demonstrating the high homogeneity of WPU-4 [41]. In addition, characterization analysis revealed an Mw of 27.30 kDa, an Mn of 14.04 kDa, and a resultant polydispersity index (PDI) of 1.94 for this polysaccharide fraction (Table 1). The combination of a narrow Mw profile and a near 1 PDI indicated that the polysaccharide component WPU-4 could be categorized as a macromolecule with moderate dispersity [52]. A study has reported that the controlled and precise shearing effect of ultrasound can reduce the differences among polysaccharides of various Mw [9], leading to a narrower Mw distribution and improved uniformity of the polysaccharides.

**Table 1:** Molecular weight determination results of WPU-4.

Sample	Mn (kDa)	Mw (kDa)	Mw/Mn (PDI)
WPU-4	14.04	27.30	1.94

Fig. 4B depicts the relationship between molecular radius and molar mass, and the slope value is found to indicate the molecular configuration of biopolymers [53]. The curve plotted as a correlation for RMS (*R_g_*) against molar mass (*M*) presented a nearly U-shaped profile. The corresponding calibration curve equation obtained from linear regression is:

(5) $${\text{log}}_{\text{1}0}\left(R_{g}\right)=-0.0\text{35}\;+0.{\text{28log}}_{\text{1}0}\left(M\right)$$

The slope of the regression line was 0.28 ± 0.03, confirming that WPU-4 possesses a compact, cross-linked, and branched structure [18].

#### 3.7.2 Fourier Transforms Infrared Spectra (FTIR) of WPU-4

To analyze the functional groups in the WPU-4, FTIR was employed with a wavenumber range of 4000–400 cm^−1^ (Fig. 4C). Five characteristic absorption band regions (4000–2500 cm^−1^, Region I; 1800–1500 cm^−1^, Region II; 1500–1200 cm^−1^, Region III; 1200–800 cm^−1^, Region IV; < 800 cm^−1^, Region V) were detected, confirming that the analyzed substance was a polysaccharide [54]. Specifically, the broad and prominent peak at 3390.9 cm^−1^ is attributable to hydroxyl group stretching vibration, which is a typical characteristic of polysaccharides [53]. The absorption at 2928.3 cm^−1^ corresponds with the stretching vibration of C–H in polysaccharides [55]. The absorption at 1422.8 cm^−1^ was characteristic of C–O stretching vibration [56]. The absorptions at 1152.7 cm^−1^ and 1020.5 cm^−1^ indicated the asymmetric stretching vibration of C–O–C, demonstrating the existence of pyranose groups and α–glycosidic linkages [52]. In addition, the region of 911.4 cm^−1^ suggested the asymmetric vibration of glucopyranose rings [57]. The characteristic peak at 767.7 cm^−1^ was correlated with C–C bonds [58].

#### 3.7.3 Monosaccharide composition analysis of WPU-4

The monosaccharide composition of WPU-4 is presented in Fig. 4D. The primary monosaccharide component of WPU-4 was glucose (Glu) (97.46 mol%), followed by mannose (Man) at 2.54 mol%. As the predominant monosaccharide, Glu accounted for over 90 mol%, implying that Glu constituted the major constituent of the polysaccharide backbone [59]. As reported by Lucena et al. [7], the exopolysaccharide extracted from water kefir grains using hot water extraction (EPSwk) was predominantly composed of Glu, accounting for 98 mol%. The subtle differences may be attributed to the fact that cavitation and shear forces induced by ultrasound cause scission of the polysaccharide chains and glycosidic bond hydrolysis, thereby modifying the monosaccharide composition of the polysaccharides [60].

#### 3.7.4 Methylation analysis

For further elucidation of the structural information of WPU-4, the linkage pattern and content of its sugar residues were measured. Table 2 illustrates that the methylation results of WPU-4 exhibited six substitution types, including t-Glcp-(1→ (15.33%), →3)-Manp-(1→ (1.33%), →6)-Glcp-(1→ (72.33%), →3,6)-Glcp-(1→ (7.80%), →4,6)-Glcp-(1→ (1.43%), and → 2,6)-Glcp-(1→ (1.78%). Therefore, the backbone was determined to be predominantly composed of 1,6-linked glucose, with the terminal positions being occupied by t-Glcp-(1 → . Meanwhile, the glucose residues in the backbone formed branching structures at position *O*3 (→3,6)-Glcp-(1 → )), *O*4 (→4,6)-Glcp-(1 → )), and *O*2 (→2,6)-Glcp-(1 → )). And it was mainly branched at position *O*3. This structural feature aligned with the findings of Fels et al. [5]. Additionally, a trace amount of → 3)-Manp-(1 → was detected, which was presumed to be a component of the polysaccharide's branched structure.

**Table 2:** Methylation analysis data for WPU-4.

Linkage pattern	Methylated sugar	Mass fragments (*m*/*z*)	RT (min)	Molar ratio (%)
t-Glcp-(1→	2,3,4,6-Me_4_-Glcp	87,102,118,129,145,161,162,205	11.994	15.33
→3)-Manp-(1→	2,4,6-Me_3_-Manp	87,101,118,129,16,202,234	16.033	1.33
→6)-Glcp-(1→	2,3,4-Me_3_-Glcp	87,99,102,118,129,162,189,233	18.302	72.33
→3,6)-Glcp-(1→	2,4-Me_2_-Glcp	87,101,118,129,18,202,234	23.293	7.80
→4,6)-Glcp-(1→	2,3-Me_2_-Glcp	85,102,118,127,15,162,201,261	23.439	1.43
2,6)-Glcp-(1→	3,4-Me_2_-Glcp	87,88,99,100,129,130,189,190	24.072	1.78
				100

#### 3.7.5 SEM

The microstructure of WPU-4 was characterized using a scanning electron microscope (SEM). Fig. 4E-G presented the SEM images of WPU-4 at magnification levels of 200× (Fig. 4E), 500× (Fig. 4F), and 1000× (Fig. 4G). As observed from the images, WPU-4 exhibited a sheet-like structure with relatively large porous features. Additionally, WPU-4 was found to have an uneven, loose, and porous network structure on its surface, and its interior showed a honeycomb-like morphology. It was hypothesized that such structural characteristics might enhance the solubility of polysaccharides and affect their bioactivity [18]. Unlike the previous findings by Lucena et al. [7], the results suggested that the surface of EPSwk was rough and irregular with very few pores. The distinct structural features of WPU-4 are likely attributable to the sustained cavitation effect, high pressure, and turbulent shear forces induced during UE [61]. Chen et al. [38] found that different extraction approaches affect the branching structure and crosslinking network of the polysaccharides, with the ultrasonically extracted polysaccharides from Bletilla striata exhibiting a regular lamellar structure and numerous large cavity structures.

### 3.8 Effect of WPU on the biological activity of GY

**Fig. 5:**
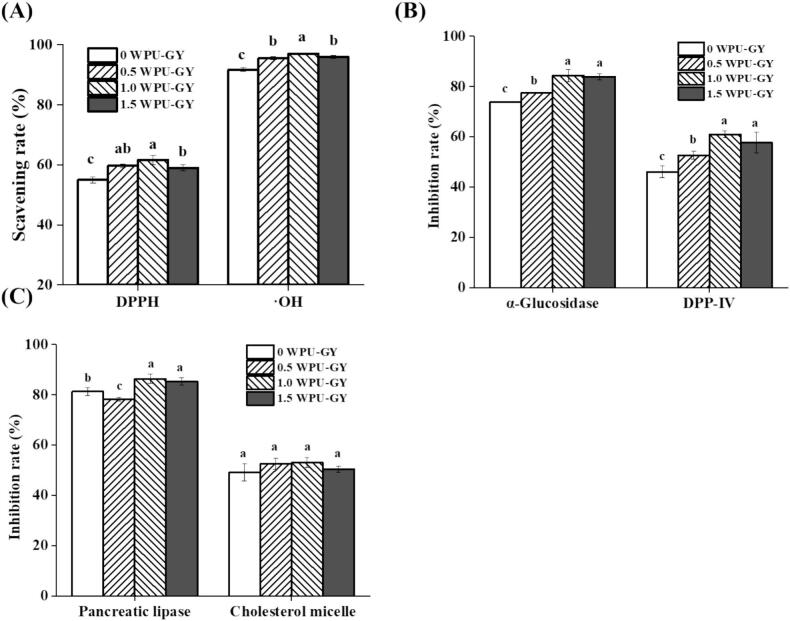
Antioxidant (A), antidiabetic (B), and hypolipidemic (C) activities of WPU-supplemented goat yogurt at different concentrations. Note: Different lowercase letters (a, b, c) denote significant differences (*p* < 0.05).

Fig. 5A illustrates that after the addition of WPU, both the DPPH and ·OH scavenging rates of GY increased significantly (*p* < 0.05). Moreover, 1.0WPU-GY exhibited the optimal DPPH and ·OH scavenging rates, which were 61.67% and 97.09%, respectively. The enhanced antioxidant activity might be attributed to the inherent antioxidant activity of WPU, as well as its ability to promote strains' fermentation after addition, which facilitated the production of additional antioxidant substances, such as bioactive peptides and organic acids [62], [63]. Additionally, it has been reported that polysaccharides can interact with casein micelles to form a continuous and dense gel network, reduce the pore size of the system, and inhibit protein hydrolysis, thereby protecting bioactive components from structural damage and maintaining their functional stability [64]. Consistent with this finding, Lin et al. [65] also clearly demonstrated that the incorporation of *Tremella fuciformis* polysaccharides (TFPS) enhanced the antioxidant activity of nutty plant-based yogurt, further confirming that polysaccharides supplementation is a useful strategy to augment the antioxidant capacity of fermented milk.

In comparison with the 0WPU-GY, GY with added WPU displayed a significant enhancement (*p* < 0.05) in α-Glu and DPP-IV inhibition (Fig. 5B). Additionally, 1.0WPU-GY and 1.5WPU-GY demonstrate no significant difference (*p* > 0.05). This indicated that GY supplied with WPU possesses the ability to inhibit both α-Glu and DPP-IV. Currently, the specific mechanism underlying the antidiabetic activity of WPU is not fully understood and requires further investigation via *in vivo* experiments in the future. Fig. 5C illustrates the effects of WPU with different concentrations on the PL and MC inhibition rates of GY. When the addition of WPU was 1.0 mg/mL and 1.5 mg/mL, the PL inhibition activity was significantly augmented (*p* < 0.05), reaching 86.43% and 85.39%, respectively. The inhibitory effect of WPU on PL might be associated with its extraction method. Ren et al. [66] reported that ultrasound-assisted extraction of *Lonicera macranthoides* polysaccharides (UAE-LMP) resulted in a PL inhibition rate of 38.96%, whereas compound enzymatic extracted LMP (CE-LMP) exhibited a rate of only 13.33%. This indicates that ultrasound-assisted extraction could significantly enhance the inhibitory activity of polysaccharides against lipases, a phenomenon attributed to the improved structural properties and oil-holding capacity of polysaccharides obtained via ultrasound extraction. However, the addition of WPU (0.5–1.5 mg/mL) showed no significant influence on the MC inhibition rate. A plausible explanation is that GY inherently contains casein micelles and lactic acid fermentation metabolites, which already provide a basal inhibition of MC. Thus, the addition of WPUs did not lead to synergistic enhancement, resulting in no significant change in MC inhibition rate. Additionally, Song et al. [67] demonstrated that *Flammulina velutipes* polysaccharide combined with fermented milk could regulate dyslipidemia through the PI3K/Akt signaling pathway, indicating the synergistic antihyperlipidemic potential of fermented milk and polysaccharide complexes. In summary, when the addition of WPU was 1.0 mg/mL, the GY exhibited the optimal antioxidant, hypoglycemic, and hypolipidemic activities. Therefore, 1.0WPU-GY was selected for further study of storage stability.

### 3.9 Storage stability of GY during cold storage

#### 3.9.1 Physicochemical properties determination

**Fig. 6:**
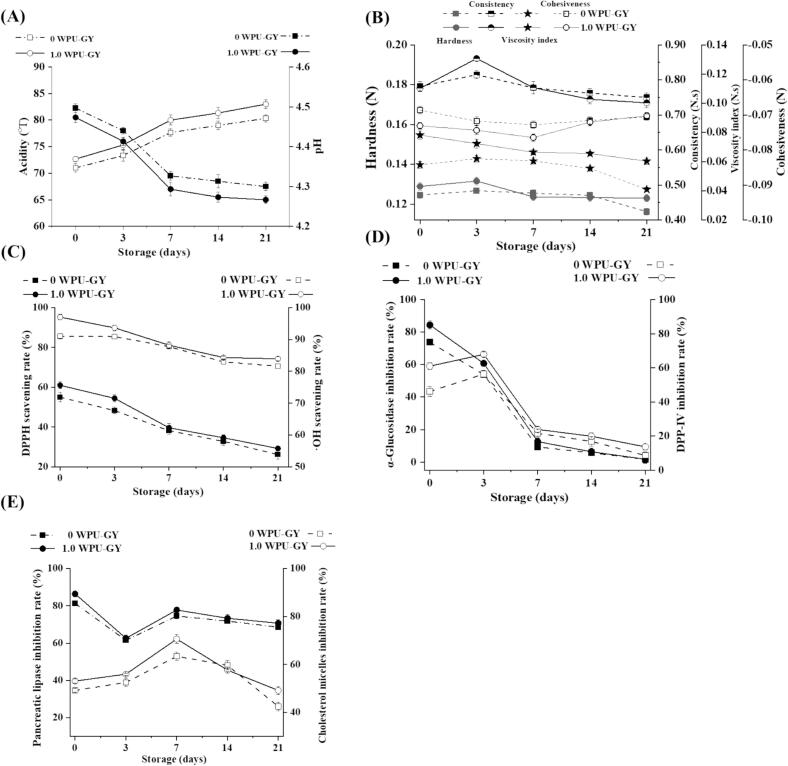
Changes in pH and titratable acidity (A), texture (B), antioxidant (C), antidiabetic (D), and hypolipidemic (E) activities of goat yogurt in 21-day storage.

The TA and pH of 0WPU-GY and 1.0WPU-GY during the storage period are shown in Fig. 6A. Post 21-day storage at 4 °C, 1.0WPU-GY showed a lower pH and a higher TA compared with 0WPU-GY. A reduction in pH value was indicative of elevated organic acid production, such as lactic acid, during the fermentation process [68], which suggested that WPU could promote microbial fermentation. Lin et al. [65] found that adding TFPS to nutty plant-based yogurt (NPBY) could increase the activity of lactic acid bacteria (LAB), thereby leading to a reduction in pH.

The changes in hardness, consistency, viscosity index, and cohesiveness of 0WPU-GY and 1.0WPU-GY throughout a 21-day storage period are shown in Fig. 6B. After the 21-day storage, the hardness (0.12 N) and viscosity index (0.060 N.s) of 1.0WPU-GY were higher than those of 0WPU-GY, respectively. One potential contributing factor might be WPU itself, which plays an effective role as a thickening agent. Additionally, it was reported that polysaccharides interacted with casein, enhanced protein aggregation, and increased the viscosity and adhesiveness of fermented milk [69]. thereby contributing to the overall hardness and viscosity.

#### 3.9.2 Antioxidant activities

The changes in antioxidant activity of GY during the 21-day storage duration are shown in Fig. 6C. The DPPH and ·OH scavenging activities of 0WPU-GY and 1.0WPU-GY decreased slightly with the extension of storage time. A decline in pH during storage, which led to a reduction in the active substances with antioxidant capacity in samples, might also be one of the contributing factors [70]. In addition, 1.0WPU-GY consistently showed higher antioxidant activity than 0WPU-GY. Consistent with the findings of Li et al. [13], the antioxidant activity of set fat-free goat yogurt supplemented with Ganoderma leucocontextum polysaccharide exhibited a significant decreasing trend during 21 days at 4 °C, with a smaller decline compared to the non-supplemented control group. Zhong et al. [71] found that when *Exidia yadongensis* polysaccharide (EYP) was added to fat-free stirred mango buffalo yogurt (PMY), the yogurt exhibited higher DPPH and ·OH scavenging rates during the 25-day storage period at 4 °C, in comparison with CMY (the yogurt without EYP). Shi et al. [70] also found that the antioxidant activity of neoagaroligosaccharides (NAOs)-added yogurt was significantly improved.

#### 3.9.3 Antidiabetic activities

During 21-day storage at 4 °C, the α-Glu and DPP-IV inhibition activities of 0WPU-GY and 1.0WPU-GY were compared, with results presented in Fig. 6D. Throughout 21 days of storage, the α-Glu inhibition rate of 1.0WPU-GY remained consistently higher than that of 0WPU-GY. Additionally, a tendency for first increase and then decline in DPP-IV inhibition rate was discovered for both samples. On day 3 of the storage period, the DPP-IV inhibition rates of 0WPU-GY and 1.0WPU-GY peaked at 56.37% and 67.90%, respectively. Notably, the inhibition rate against DPP-IV of 1.0WPU-GY was also consistently higher than that of 0WPU-GY over the entire 21-day storage period. At the 21st day, the DPP-IV inhibition rate of 1.0WPU-GY was maintained at 13.75%, while that of 0WPU-GY dropped to 8.68%. The results showed that WPU could improve the retention of GY antidiabetic activity during storage. Ramchandran and Shah [14] found that yogurt produced with an EPS-producing strain and 3% (w/v) inulin exhibited antidiabetic potential over the 0–14-day storage period. In contrast, yogurt containing a non-EPS-producing strain showed no antidiabetic activity.

#### 3.9.4 Hypolipidemic activities

The inhibitory activities of 0WPU-GY and 1.0WPU-GY on PL and MC were evaluated over a 21-day storage period, as demonstrated in Fig. 6E. The PL inhibition activities of 0WPU-GY and 1.0WPU-GY were the highest on day 0 of storage, followed by a decrease on day 3. On day 7, they slightly increased to 74.55% and 77.77%, respectively, before continuing to decrease slightly thereafter. By day 21 of storage, the PL inhibition rates of the 0WPU-GY and 1.0WPU-GY reached 68.59%, 70.70%, respectively. This result indicated that 0WPU-GY and 1.0WPU-GY maintained a high PL inhibition rate during storage. Both 0WPU-GY and 1.0WPU-GY exhibited a trend where MC inhibition rates first increased and then declined throughout the storage period. The peak values reached on the 7th day of storage were 63.41% and 70.60%, respectively. Moreover, the MC inhibition rate of 1.0WPU-GY was 15.75% greater than that of 0WPU-GY after the 21-day storage. Overall, both samples exhibited excellent inhibition rates of PL and MC during 21-day storage. Overall, adding 1.0 mg/mL WPU to GY was a feasible method to enhance the maintenance of hypolipidemic activity.

## 4 Conclusion

This study aimed to develop an efficient UE method for extracting WPU and explore its application in goat yogurt. SF experiments combined with the RS methodology were employed to optimize the UE conditions of extracting WPU. The optimal UE conditions were confirmed as 340 W, 42 min, 20 mL/g, and 80 °C, under which the WPU yield reached 27.64%. Structural characterization showed that the purified fraction WPU-4 was glucose-dominant with a compact branched structure. While *in vitro* functional assays demonstrated WPU's superior bioactivities. And it was determined that 1.0 mg/mL was the optimal WPU addition for GY, which significantly enhanced GY's antioxidant, antidiabetic, and hypolipidemic activities. These effects remained prominent in the sample after 21 days of storage compared to the 0WPU-GY. In summary, the clarification of WPU’s properties and functions and application in GY has introduced fresh prospects for the food industry. Despite the clarification of the optimized extraction process, structural characteristics, and functional activities of WPU in GY, as well as the confirmation of its stable enhancing effects during storage, the present study has certain limitations. Functional evaluations of WPU were primarily conducted through *in vitro* assays, with no *in vivo* experiments. Meanwhile, the specific molecular mechanisms underlying the hypoglycemic and hypolipidemic activities of WPU, as well as its interactions with key metabolic pathways in the body, remain incompletely elucidated. Future research is warranted to combine *in vivo* experiments to clarify these underlying mechanisms.

## CRediT authorship contribution statement

**Wenjuan Zhang:** Writing – original draft, Validation, Software, Methodology. **Guowei Shu:** Writing – review & editing, Supervision, Funding acquisition, Conceptualization. **Zongcai Zhang:** Software, Investigation. **Ting Li:** Formal analysis, Data curation. **Huan Lei:** Resources, Project administration. **Huayang Xue:** Investigation, Funding acquisition. **Zhi Wang:** Methodology, Funding acquisition. **Xiaolin Yao:** Methodology, Formal analysis. **Guoliang Li:** Supervision, Investigation.

## Declaration of competing interest

The authors declare that they have no known competing financial interests or personal relationships that could have appeared to influence the work reported in this paper.

## Data Availability

Data will be made available on request.
