# Supplementary material for: Water kefir grain polysaccharides: Ultrasonic-assisted extraction optimization, structural characterization, bioactivities, and application in goat yogurt
Source: Ultrason Sonochem. 2026 Mar 18;128:107827. doi: 10.1016/j.ultsonch.2026.107827 (PMC13022633; doi:10.1016/j.ultsonch.2026.107827)

**Supplementary Material**

**Water kefir grain polysaccharides: Ultrasonic-assisted extraction optimization, structural characterization, bioactivities, and application in goat yogurt**

Wenjuan Zhang^a^, Guowei Shu^a, *^, Zongcai Zhang^a^, Ting Li^a^, Huan Lei^b^, Huayang Xue^c^, Zhi Wang^c^, Xiaolin Yao^a^, Guoliang Li^a, *^

^a^ School of Food Science and Engineering, Shaanxi University of Science and Technology, Xi’an 710021, China

^b^ Xi’an Baiyue Gaot Milk Corp., Ltd., Xi’an 710089, China

^c^ Shaanxi Youlishi Dairy Group Co., Ltd., Xianyang, 713300, China

*Corresponding author

E-mail addresses: shuguowei@gmail.com (G. Shu) and 61254368@163.com (G. Li)

**Table S1 The factors of the Box-Behnken test and their levels**

| Level | *A*: Ultrasonic power (W) | *B*: Ultrasonic time (min) | *C*: Liquid-to-solid ratio (mL/g) |
| --- | --- | --- | --- |
| 1 | 280 | 30 | 10 |
| 0 | 320 | 40 | 15 |
| -1 | 360 | 50 | 20 |

**Table S2 Experimental design and results of Box-Behnken**

| No. | *A*:  Ultrasonic power (W) | *B*:  Ultrasonic time (min) | *C*:  Liquid-to-solid ratio (mL/g) | Yields (%) |
| --- | --- | --- | --- | --- |
| 1 | 280 | 30 | 15 | 15.87 |
| 2 | 320 | 50 | 20 | 25.01 |
| 3 | 320 | 50 | 10 | 16.42 |
| 4 | 360 | 40 | 20 | 25.39 |
| 5 | 320 | 40 | 15 | 24.94 |
| 6 | 320 | 40 | 15 | 26.99 |
| 7 | 360 | 50 | 15 | 19.13 |
| 8 | 320 | 30 | 20 | 19.89 |
| 9 | 280 | 50 | 15 | 13.36 |
| 10 | 280 | 40 | 20 | 16.60 |
| 11 | 320 | 30 | 10 | 21.35 |
| 12 | 280 | 40 | 10 | 18.66 |
| 13 | 320 | 40 | 15 | 24.74 |
| 14 | 360 | 30 | 15 | 22.24 |
| 15 | 360 | 40 | 10 | 25.18 |

**Table S3 Regression model analysis of variance**

| Source | Sum of Squares | df | Mean Square | *F*-value | *P*-value |
| --- | --- | --- | --- | --- | --- |
| Model | 236.41 | 9 | 26.27 | 7.04 | 0.0224* |
| *A-* | 94.19 | 1 | 94.19 | 25.24 | 0.0040** |
| *B-* | 3.69 | 1 | 3.69 | 0.9877 | 0.3659 |
| *C-* | 3.48 | 1 | 3.48 | 0.9339 | 0.3782 |
| *AB* | 0.09 | 1 | 0.09 | 0.0241 | 0.8827 |
| *AC* | 1.29 | 1 | 1.29 | 0.3452 | 0.5824 |
| *BC* | 25.25 | 1 | 25.25 | 6.77 | 0.0482* |
| *A^2^* | 46.75 | 1 | 46.75 | 12.53 | 0.0166* |
| *B^2^* | 69.81 | 1 | 69.81 | 18.71 | 0.0075** |
| *C^2^* | 1.08 | 1 | 1.08 | 0.2894 | 0.6137 |
| Residual | 18.66 | 5 | 3.73 |  |  |
| Lack of Fit | 15.56 | 3 | 5.19 | 3.34 | 0.2387 |
| Pure Error | 3.10 | 2 | 1.55 |  |  |
| Cor Total | 255.06 | 14 |  |  |  |

*Denotes a significant difference with *p* < 0.05.

** Denotes a significant difference with *p* < 0.01.

**Figure S1** GC-MS analysis of partially methylated alditol acetate (PMAA) derivatives of WPU-4. (A) Total ion chromatogram (TIC) of PMAA derivatives separated by gas chromatography. (B–G) mass spectra of identified PMAA derivatives: (B) 2,3,4,6-Me₄-Glcp, (C) 2,4,6-Me₃-Manp, (D) 2,3,4-Me₃-Glcp, (E) 2,4-Me₂-Glcp, (F) 2,3-Me₂-Glcp, and (G) 3,4-Me₂-Glcp.


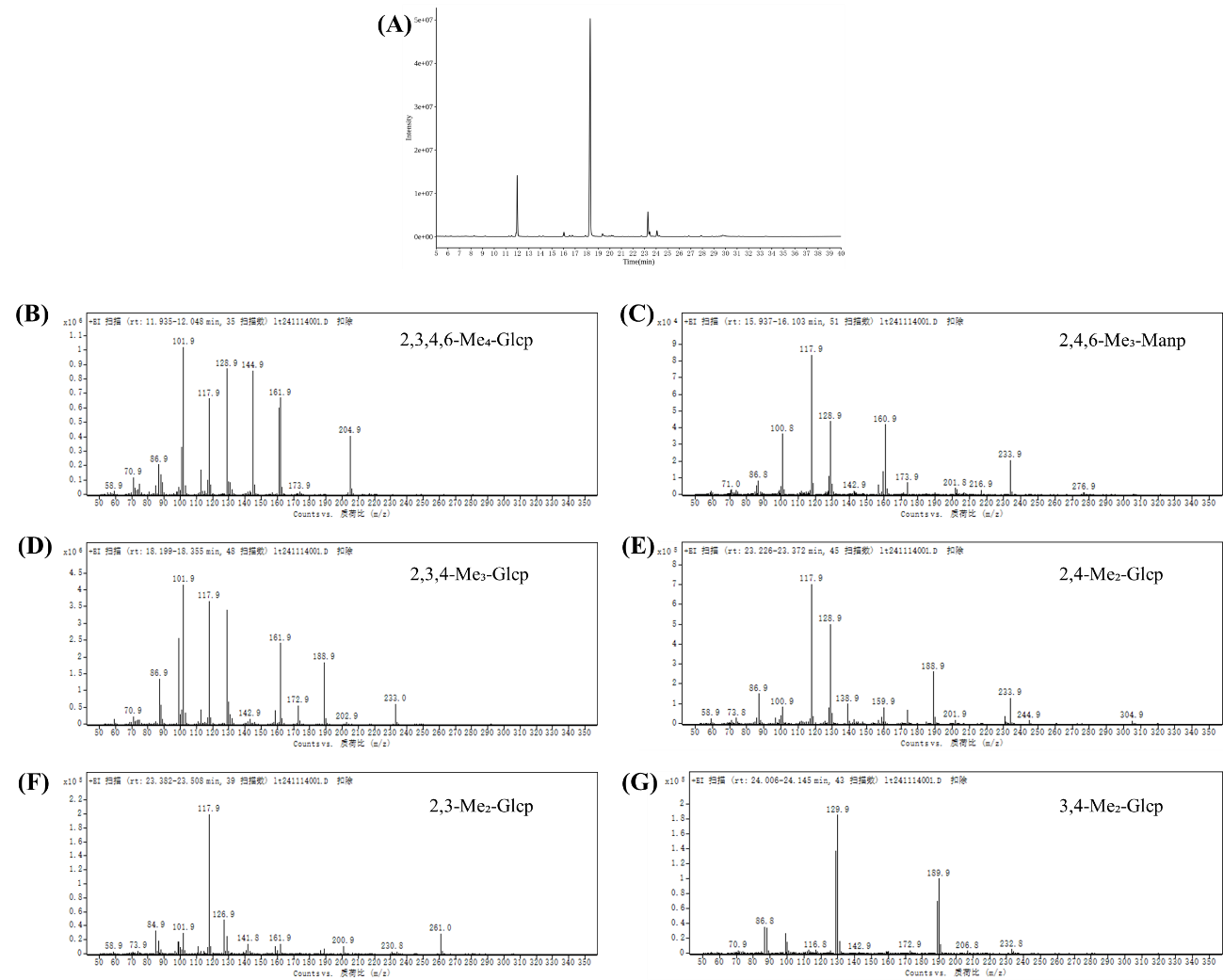

Supplement: Supplementary Data 1 [file mmc1.docx]
